# Qualitative evaluation of a package of implementation strategies codesigned to support the introduction of multiple micronutrient supplementation (MMS) for pregnant women in Bamako, Mali

**DOI:** 10.1111/mcn.13712

**Published:** 2024-08-22

**Authors:** Aissata Ba, Monica J. Fox, Adama Mamby Keita, Kristen M. Hurley, Shannon E. King, Samba Sow, Kounandji Diarra, Mahamane Djiteye, Baba Seydou Kanté, Moussa Coulibaly, Ousmane Dembele, Lisa M. Noguchi, Pooja Sripad, Peter J. Winch

**Affiliations:** ^1^ Jhpiego Bamako Mali; ^2^ Department of International Health Johns Hopkins Bloomberg School of Public Health Baltimore Maryland USA; ^3^ Vitamin Angel Alliance Goleta California USA; ^4^ Center for Vaccine Development Bamako Mali; ^5^ Jhpiego Baltimore Maryland USA

**Keywords:** adherence, antenatal care, counselling, iron and folic acid, Mali, multiple micronutrient supplementation, pregnant women

## Abstract

Mali national policy recommends that women take iron and folic acid supplements (IFA) from the time of the first antenatal care (ANC) visit, throughout pregnancy and during the first 3 months after delivery. In 2020, the World Health Organization (WHO) updated their ANC guidelines to recommend the United Nations International Multiple Micronutrient Antenatal Preparation (UNIMMAP) formulation of multiple micronutrient supplements (MMS) in the context of rigorous research, including implementation research. In Bamako, Mali, a codesign process was used to tailor antenatal care MMS packaging and counselling materials aimed at optimizing delivery and uptake of and adherence to MMS. This paper presents the codesign process along with the results of a post‐intervention qualitative assessment to evaluate the behaviour change intervention. At the conclusion of the intervention, we conducted semistructured qualitative interviews with 24 women who had received the intervention and six pharmacy managers from the six health centres participating in the study. We conducted two focus groups with midwives who had delivered the intervention and two group discussions with family members of women who had received the intervention. Respondent perspectives reveal an easy experience transitioning from previously used IFA. Women and providers concur that the intervention counselling materials and visual aids were instrumental in influencing the perceived benefit and uptake of MMS. Family members play an influential role in pregnant women's decision‐making regarding MMS uptake. MMS and the associated implementation strategies developed through the codesign process were found to be a highly acceptable intervention.

## Key messages

- United Nations International Multiple Micronutrient Antenatal Preparation (UNIMMAP) multiple micronutrient supplementation (MMS) and the associated implementation strategies developed through the codesign process were found to be highly acceptable interventions.
- Any national transition from iron and folic acid supplements to MMS will need to include planning and budgeting for necessary resources, training on antenatal care MMS counselling and ongoing supportive supervision to antenatal care providers.
- Additional implementation strategies might focus on community‐based behaviour change strategies that support the uptake of and adherence to MMS as well as supply chain mechanisms to support a reliable and sustainable stock of UNIMMAP MMS.

## Graphical abstract

Pregnant women, midwives and others participated in a codesign process to develop implementation strategies to support the delivery of a multiple micronutrient supplementation (MMS) intervention. Both the MMS and the accompanying strategies were found to be highly acceptable to pregnant women and midwives in Bamako Mali.

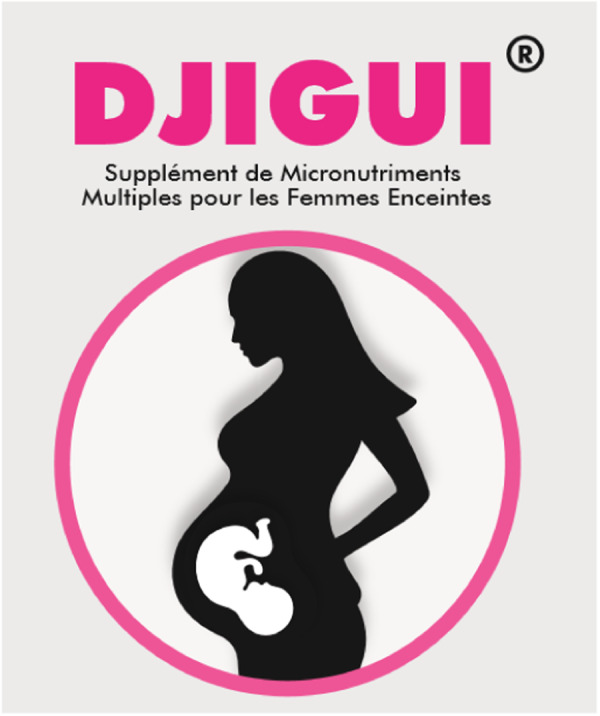

## 1 INTRODUCTION

### 1.1 Multiple micronutrient supplementation in pregnancy in Mali

Anaemia is prevalent among women of reproductive age (WRA) in low‐ and middle‐income countries (Bourassa et al., 2019). Causes of anaemia include parasitic infections, chronic infections, inflammation, inherited disorders, gynaecological and obstetric conditions, and deficiencies in folate, riboflavin and vitamins A and B12. However, iron deficiency, primarily due to inadequate dietary iron intake, is considered the most common nutritional deficiency leading to anaemia (Brittenham et al., 2023). To address maternal anaemia in Mali, the national policy recommends that women take iron‐folic acid (IFA) from the time of the first antenatal care (ANC) visit, throughout pregnancy and during the first 3 months after delivery.

At the global level, two decades of rigorous evidence have shown that compared to IFA supplements alone, multiple micronutrient supplements (MMS) result in continued prevention of maternal anaemia (Gomes et al., 2022) and greater reductions in low birthweight, pre‐term and small for gestational age infants (Bourassa et al., 2019; Keats et al., 2019; Smith et al., 2017), especially among anaemic and underweight women (Smith et al., 2017). Following a review of available evidence, the World Health Organization (WHO) updated the *WHO recommendations on antenatal care for a positive pregnancy experience* in 2020 to recommend the United Nations International Multiple Micronutrient Antenatal Preparation (UNIMMAP) formulation of MMS in the context of rigorous research, including implementation research that informs MMS introduction by investigating the acceptability, feasibility, cost‐effectiveness and equity of implementing the MMS intervention (World Health Organization, 2020).

In Mali, the prevalence of chronic energy deficiency or underweight among WRA was 8.5% in September 2021 (World Food Program, 2023). The 2018 Mali Demographic and Health Survey found that 63% of WRA are anaemic (haemoglobin less than 12 g/dL) and 4% are severely anaemic (haemoglobin less than 7 g/dL) (INSTAT, 2019). Furthermore, less than half of pregnant women attended ANC‐1 ([36%] first visit) during their first trimester or took iron supplements (28%) for at least 90 days during pregnancy (INSTAT, 2019). Hence, most pregnant women start IFA late and do not continue supplementation throughout their pregnancy, contributing to the high rates of maternal anaemia. Despite the persistence of low adherence to iron supplements, previous evidence from a small study in Mali (*n* = 70) indicates that pregnant women do adhere well to micronutrient supplements when they are provided with consistent and easily understandable information and counselling (Aguayo et al., 2005). This study sought to take a closer look at the factors affecting adherence, including the novel counselling provided to them by midwives in a larger sample of women in a greater number of health facilities. Despite evidence for the benefits of MMS (Bourassa et al., 2019; Caniglia et al., 2022; Keats et al., 2019), calls for a change in recommendations (Bourassa et al., 2020) and prior research demonstrating the acceptability of MMS in Mali (Aguayo et al., 2005), the potential benefits of micronutrient supplementation for pregnant women and their infants will only be realized through more effective programming aimed at increasing maternal adherence to supplementation.

To inform ANC delivery strategies to increase adherence to antenatal MMS, Jhpiego in partnership with the Center for Vaccine Development in Mali (CVD‐Mali), Mali Ministry of Health and the Johns Hopkins Bloomberg School of Public Health (BSPH)—Center for Human Nutrition conducted implementation research to compare adherence to and acceptability of three different delivery approaches to supplementation in pregnancy.

The comparison was conducted across 6 health facilities in and around Bamako which were randomly assigned to the following intervention groups: (1) standard of care (IFA tablets dispensed in 30‐count blister packs distributed monthly); (2) UNIMMAP MMS tablets dispensed in 30‐count bottles distributed monthly along with an enhanced counselling strategy or (3) UNIMMAP MMS tablets dispensed in one 180‐count bottle along with an enhanced counselling strategy.

### 1.2 Adherence to antenatal multiple micronutrient supplementation

Many studies have examined factors affecting adherence to antenatal multiple micronutrient supplementation. Surveys based on population‐based samples have demonstrated associations between adherence to antenatal supplementation and maternal age (Getachew et al., 2018; Wana, 2020), maternal education (Getachew et al., 2018; Maina‐Gathigi et al., 2013; Wana, 2020), wealth index (Getachew et al., 2018; Maina‐Gathigi et al., 2013; Wana, 2020), distance to health facility (Getachew et al., 2018; Wana, 2020), urban residence (Demis et al., 2019; Nisar et al., 2014), number of ANC visits during pregnancy (Demis et al., 2019; Gebremedhin et al., 2014; Getachew et al., 2018; Wana, 2020), the timing of first ANC visit (Demis et al., 2019; Getachew et al., 2018), receiving information about antenatal supplementation (Demis et al., 2019; Gebremedhin et al., 2014; Getachew et al., 2018) and maternal anaemia (Demis et al., 2019; Gebremedhin et al., 2014; Getachew et al., 2018; Wana, 2020).

Qualitative and mixed methods have identified an even broader range of factors affecting adherence to antenatal micronutrient supplementation. Table 1 summarizes these findings, categorized into two types of factors: (1) factors related to the functioning of the health system and utilization of health services and (2) psychosocial factors based on constructs in the Health Belief Model (Jones et al., 2015).

**Table 1:** Factors affecting adherence with antenatal supplementation from qualitative, formative and mixed methods studies.

Factors associated with health system functioning and utilization of health services
Availability of ANC, distance to health facilities (Galloway et al., 2002; Kamau, 2020; Siekmans et al., 2018; Simuyemba et al., 2020) Late initiation of ANC (after the first trimester) (Demis et al., 2019; Galloway et al., 2002; Kamau, 2020; Siekmans et al., 2018; Simuyemba et al., 2020; Tinago et al., 2017; Wana, 2020) Number of ANC visits made during pregnancy (Demis et al., 2019; Galloway et al., 2002; Getachew et al., 2018; Kamau, 2020; Siekmans et al., 2018; Simuyemba et al., 2020; Wana, 2020) Stockouts of antenatal supplements and/or supplements not provided during ANC (Galloway et al., 2002; Getachew et al., 2018; Kamau, 2020; Sambili et al., 2016; Siekmans et al., 2018; Simuyemba et al., 2020; Wana, 2020) Exposure to information, and provision of counselling on antenatal supplements during ANC (Alam et al., 2015; Demis et al., 2019; Galloway et al., 2002; Getachew et al., 2018; Mbhenyane & Cherane, 2017; Sambili et al., 2016; Siekmans et al., 2018; Simuyemba et al., 2020; Tinago et al., 2017)
Psychosocial factors from the Health Belief Model
Perceived severity of maternal anaemia and health risks from maternal anaemia (Demis et al., 2019; Galloway et al., 2002; Getachew et al., 2018; Kamau, 2020; Tinago et al., 2017; Wana, 2020) Perceived benefits of antenatal supplements ∘ Supplements, in general, the importance of taking antenatal supplements (Demis et al., 2019; Galloway et al., 2002; Getachew et al., 2018; Mbhenyane & Cherane, 2017; Tinago et al., 2017; Wana, 2020) ∘ Mother healthier, easier delivery (Aguayo et al., 2005; Alam et al., 2015; Galloway et al., 2002; Kamau, 2020; Tinago et al., 2017; Wana, 2020) ∘ Newborn healthier, fetus grows better (Aguayo et al., 2005; Alam et al., 2015; Galloway et al., 2002; Kamau, 2020; Mbhenyane & Cherane, 2017; Siekmans et al., 2018; Tinago et al., 2017; Wana, 2020)
Perceived weaknesses of antenatal supplements ∘ Side effects generally, fear of side effects (Galloway et al., 2002; Getachew et al., 2018; Mbhenyane & Cherane, 2017; Sambili et al., 2016; Siekmans et al., 2018; Simuyemba et al., 2020; Tinago et al., 2017; Wana, 2020) ∘ Nausea, vomiting, morning sickness (Aguayo et al., 2005; Alam et al., 2015; Galloway et al., 2002; Sambili et al., 2016; Simuyemba et al., 2020; Tinago et al., 2017; Wana, 2020) ∘ Increased appetite, inability to afford food (Galloway et al., 2002; Simuyemba et al., 2020; Tinago et al., 2017) ∘ Supplements will make the baby too big and delivery more difficult (Alam et al., 2015; Galloway et al., 2002; Sambili et al., 2016; Siekmans et al., 2018; Simuyemba et al., 2020; Wana, 2020) ∘ Better to avoid supplements during the first trimester (Alam et al., 2015; Galloway et al., 2002; Siekmans et al., 2018) ∘ Supplements smell or taste bad (Aguayo et al., 2005; Galloway et al., 2002; Wana, 2020)
Self‐efficacy in managing side effects (Siekmans et al., 2018) Cues to action ∘ Forgetting to take supplements is a barrier to adherence (however, strategies to remind women are rarely described) (Aguayo et al., 2005; Galloway et al., 2002; Getachew et al., 2018; Siekmans et al., 2018; Simuyemba et al., 2020; Tinago et al., 2017; Wana, 2020) ∘ Role of family members in reminding and supporting women to take supplements (Aguayo et al., 2005; Siekmans et al., 2018; Simuyemba et al., 2020; Tinago et al., 2017)

Abbreviation: ANC, antenatal care.

### 1.3 Implementation strategy development

Previous experience in Mali found that women use many terms to refer to medicines, and a single term may refer to many different medicines (Patterson et al., 2006; Searle et al., 2020). Additionally, pregnant women have difficulty distinguishing between different medicines with similar appearance (Searle et al., 2020). To differentiate UNIMMAP MMS from other medications, the study team set out to develop a distinctive name, packaging and counselling messages for MMS to be distributed during the implementation research.

### 1.4 Codesign process and results

In July 2021, six midwives, seven women who were pregnant and/or mothers of young children, two graphic designers, representatives of Jhpiego, the Ministry of Health, a local university and a local youth health advocacy group participated in a 2‐day workshop to codesign a local name, label and packaging for the MMS product and a set of counselling messages to encourage women to take MMS daily. Sample materials produced by the graphic designers after the workshop were pretested in two community health centres and one district health centre before revision and finalization.

**Figure 1:**
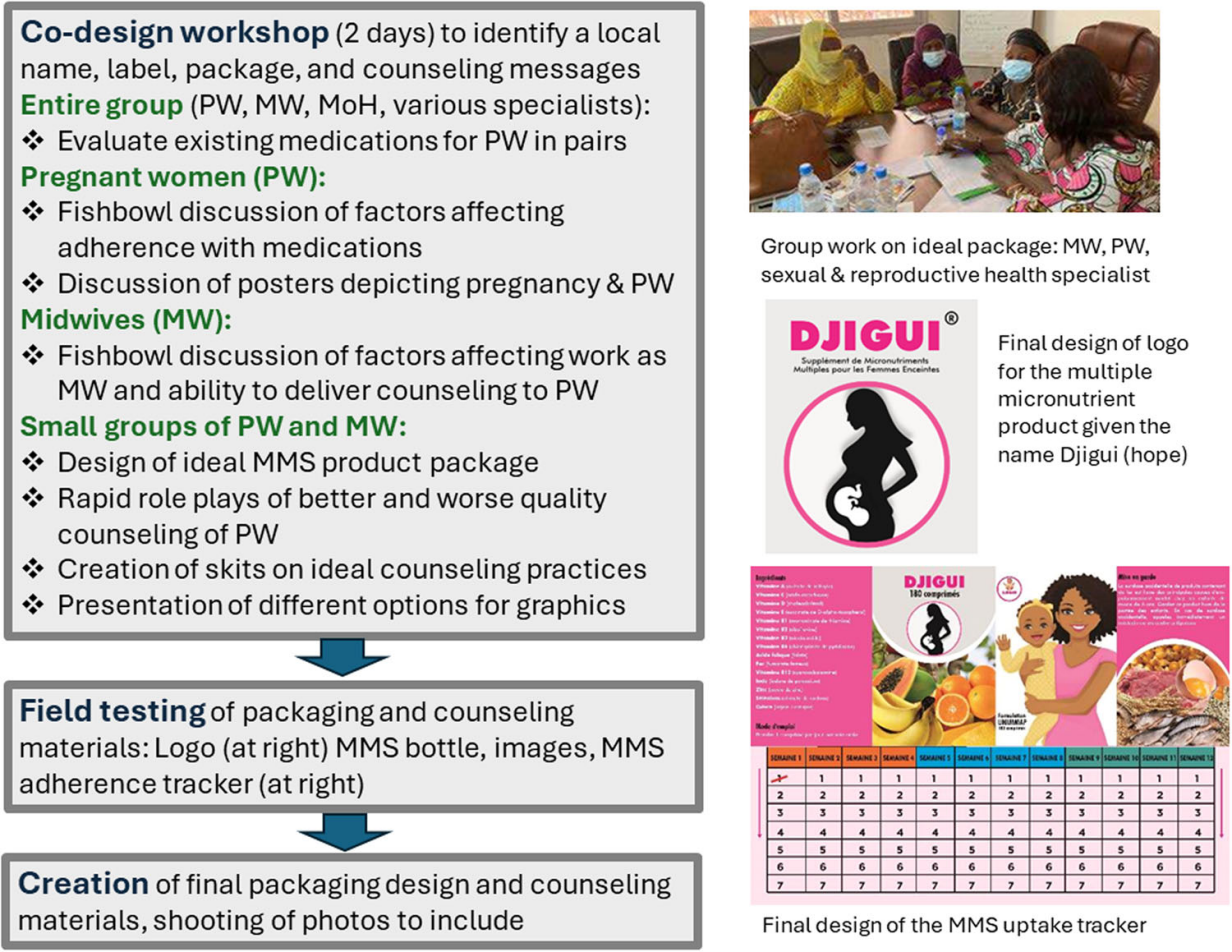
Summary of the codesign process and the materials produced. The figure describes the codesign workshop and the field testing of the materials that were produced during the codesign workshop, along with describing and providing examples of materials that were developed and used in the intervention. MMS, multiple micronutrient supplements; MoH, Ministry of Health.

The codesign process resulted in a ‘package’ of implementation strategies that midwives in the two MMS intervention groups would be trained to apply during ANC visits to support MMS uptake and adherence among pregnant women. The ‘package’ included the following: (1) logo, name and packaging of the MMS product, (2) counselling by midwives about MMS during ANC visits, (3) a counselling aid for use by midwives with photos on one side to show to women during MMS counselling and messages for midwives to share with pregnant women on the opposite side of each page that address all the psychosocial factors summarized in Table 1 and (4) a calendar for tracking daily consumption of MMS that was intended to serve as reminder to women to take the MMS. A detailed description of the codesign process, including specific workshop activities and findings from the pretesting has been described elsewhere (Ba et al., 2023; Kraemer & Olson, 2023), and Figure 1 summarizes the codesign process and the materials produced. However, key findings from the codesign process are outlined below.

#### 1.4.1 MMS product logo, name and packaging

1. Images and messages on the packaging are important for product recognition.
2. Images depicting local foods and pregnant women with relatable skin complexion and clothing facilitate understanding regardless of education.
3. Some women had concerns about vitamins inducing excessive weight gain, leading to big babies and complicated labour.

#### 1.4.2 Counselling approach and messages

1. Midwives are powerful agents that can positively affect the adherence of pregnant women.
2. ANC context necessitates brief counselling sessions.
3. Counselling should
  (a) Be positive, respectful, welcoming and judgement‐free.
  (b) Start by focusing on things women know (e.g. IFA) and progress to MMS.
  (c) Include a clear explanation of what MMS is and how it relates to a healthy pregnancy.
  (d) Include strategies for avoiding negative pregnancy outcomes.
4. A calendar can facilitate women remembering to take MMS every day.

## 2 METHODS

### 2.1 Study design

The study was conducted in January and February 2023 after the implementation of the intervention. Qualitative methods, including in‐depth interviews (IDI) and focus group discussions (FGD) were conducted in either Bambara or both Bambara and French.

### 2.2 Interviews with women

Post‐delivery semistructured IDI were conducted with 24 women exposed to the intervention, four from each of the six health centres participating in the study (eight women from each of our three study arms). Data collection was conducted between January and March 2023 with women who had been identified at their post‐delivery visit as interested in completing the IDI. Twenty‐three of the semistructured IDI were conducted in Bambara, and one was conducted in French and Bambara. The objectives of the IDI with women were to (1) understand women's perceptions of the pre‐natal supplements received during the study, (2) examine women's receipt and comprehension of counselling about the purpose and instructions for taking pre‐natal supplements, including both verbal counselling and written materials, (3) identify additional contextual factors that inhibited or encouraged adherence to the pre‐natal supplement and (4) capture any strategies women utilized or suggest for addressing challenges they or others have incurred with adhering to the pre‐natal supplement regimens.

### 2.3 Discussions with influential family members

Two group discussions were conducted with influential family members of women in the study, both in Bambara, each with six participants. The discussions with family members were similar to the interviews with women but had a stronger focus on why or why not families support MMS supplementation and its appropriate daily use. The discussions were not true focus groups but rather had a variety of participants, primarily husbands and mothers‐in‐laws of recently delivered women.

### 2.4 FGD with midwives

Two FGD, each with six participants, were conducted with midwives from the six health centres. The two FGD with midwives allowed us to gain insight from the majority (*n* = 12) of midwives implementing the intervention in our study (*n* = 15) across the six facilities. The remaining midwives were not available to participate at the time of the data collection. We conducted two FGD to make the locations for the FGD easy to reach for the midwives, due to the great distance between the participating health facilities. The language for both was a mixture of French and Bambara. One FGD consisted of midwives from the largest health centre, while the other one consisted of midwives drawn from the remaining health centres. The objectives of these FGD were to (1) understand perceptions and assess knowledge of the different pre‐natal supplements and (2) capture any strategies for addressing challenges they or others have encountered with adherence to the pre‐natal supplement regimen.

### 2.5 Discussion with pharmacy managers

Semistructured IDI were also conducted with six pharmacy managers, one from each of the six health centres with similar objectives to the FGD with the midwives. Four were conducted in Bambara only, and two in French and Bambara.

### 2.6 Data management and analysis

The IDI and FGD were semistructured, meaning that all participants were asked the same questions, and most questions had open‐ended responses that were typically 2–4 sentences in length. Some questions were closed‐ended. Recordings were transcribed and translated from Bambara into French. A database in Microsoft Access was developed for each method of data collection, with both closed‐ended and open‐ended fields or columns. Transcripts were then organized and recorded in Microsoft Access by the questions asked in the semistructured IDIs and FGD. The coding of the responses to open‐ended questions was primarily deductive. Factors affecting adherence to antenatal supplements were identified from the literature and were developed into codes. There was limited inductive coding, but several unexpected findings did emerge, which resulted in the creation of new codes.

For example, results for each module in the guide were coded and entered into the data entry form with responses to questions recorded either using numeric ratings (i.e. for more structured question and response options) or quotes (i.e. for more open‐ended responses). A summary was made of the findings from each module, following an adapted process of Framework Analysis (Gale et al., 2013; Ward et al., 2013). The interviewers entering data participated in weekly Zoom calls with the lead qualitative researcher during the period of data entry and analysis. After an initial summary of the findings document was draughted, the full qualitative team participated in an analysis workshop in Bamako to discuss each major finding and review quotes that supported each finding.

### 2.7 Ethics statement

The protocol for the study ‘Introduction of Multiple Micronutrient Supplementation to Antenatal Care in Bamako, Mali (MAMAN)’ received approval from the Ethical Review Committee of the University of Sciences, Techniques and Technologies of Bamako (USTT, B) and the Institutional Review Board of BSPH, IRB00017475. Verbal informed consent was obtained from all study participants. Investigators utilized a unique study‐generated ID for all respondents. All IDI and FGD were conducted in private locations where the risks of being over‐heard were limited. All research assistants had training in the ethical conduct of human subjects' research.

## 3 RESULTS

Six themes were identified through the qualitative assessment. The themes were (1) function and utilization of the MMS intervention package, (2) perceived benefits of taking MMS, (3) perceived benefits of MMS versus IFA, (4) perceived barriers to taking MMS, (5) other perceived barriers to taking supplements and (6) cues to action for taking MMS. The themes are discussed below.

### 3.1 Function and utilization of MMS intervention package

As described above, the MMS intervention package includes the following: (1) logo, name (Djigui) and packaging of the MMS product, (2) counselling by midwives, (3) counselling aid for midwives and (4) calendar for tracking MMS adherence. Overall, the four components were well received by women, their families, midwives and pharmacy managers.

#### 3.1.1 Logo, name (Djigui) and packaging

Through the codesign process, participants decided to name the product ‘Djigui’ instead of using the word for vitamin. Djigui is the Bambara word for ‘Hope’. Women and family members communicated high acceptance of the MMS supplement branded and packaged as ‘Djigui’. Post‐partum women interviewed recognized the potential benefits of MMS for women and children and stated that all women should be taking it.

#### 3.1.2 Counselling by midwives

During the counselling, the midwives tried to address as many of the women's concerns as possible. For example, if a woman were complaining of nausea after taking MMS, the midwife would suggest that she take it with food. Furthermore, the counselling was critical in dispelling initial doubts about MMS. However, midwives noted that one‐on‐one counselling could be difficult to conduct in the health centres given the lack of sufficient health care providers.

In addition, the health centre pharmacy managers suggested that MMS should replace IFA, but they expressed concerns regarding who should be responsible for counselling women on MMS and how to take it. Most pharmacy managers and midwives stated that midwives should first explain to the women the purpose of MMS and how to take it, but then the pharmacy managers should reinforce this information. Pharmacy managers were eager to learn more about MMS so they could provide women with more information and correctly respond to their questions.

Yes, it is necessary that pharmacists play a role in the counselling because we explain to them the importance of this drug in addition to the dosage.

—Pharmacy Manager

I think that pharmacists have a role to play in counselling especially by giving them the dosage and other techniques to encourage women to take [IFA] without problems.

—Pharmacy Manager

#### 3.1.3 Counselling aids

Midwives appreciated the 20‐card set of photos and messages developed for counselling women on the purpose of taking MMS and how to take MMS, expressing that these visual aids, together with the counselling strengthened their relationships with pregnant women. Below are two quotes from midwives regarding counselling aids.

We really appreciated the image of the midwife and the pregnant woman because the women see themselves through the image.

—Midwife

We found the counselling strategies and tools we use with the women in the study to be very good. Because there is interaction between midwives and pregnant women through the image which facilitates their understanding.

—Midwife

Similarly, women expressed appreciation for the counselling by midwives and the explanations about MMS.

When I went for my ANC, the midwife gave me information about Djigui [MMS], that it is a drug that is rich in vitamins, showing me the pictures of the foods on the bottle, and I accepted.

—Woman in one of the MMS intervention groups

Some women identified specific problems with some of the photos in the visual aids, including babies that were too old and bedrooms that did not accurately reflect the economic status of the women interviewed. The image of a woman vomiting led some women to believe that vomiting is a side effect of MMS.

In addition to the four components of the intervention package, midwives also recommended widespread promotion of MMS to all members of the community for the purpose of combatting hesitation or concerns about the intervention, arguing that building broader public awareness is key to effectively promoting adherence to MMS.

We must increase awareness on TV and everywhere in the community so that people know what Djigui [MMS] has been. So, if the understanding becomes total, people will help pregnant women to take Djigui [MMS].

—Midwife

It is to strengthen communication on the advantages of Djigui [MMS] with the community, men as well as women, even in mosques.

—Midwife

### 3.2 Perceived benefits of taking MMS supplements

Both women and their husbands recognized the benefits of MMS over IFA. Actual and/or perceived benefits commonly cited were better health and nutrition, strengthened blood, increased maternal appetite and weight gain and healthier babies. Examples of statements from women and their husbands are below.

Djigui [MMS] is a drug that is good for the health of mother and child, and in addition Djigui [MMS] strengthens the blood.

—Woman in one of the MMS intervention groups

At first, I had no appetite, but since I started taking Djigui [MMS], I have had an appetite, and I have eaten a lot. With Djigui [MMS] we avoid malformation, and it strengthens the health of the child.

—Woman in one of the MMS intervention groups

At the beginning, the pregnancy made me tired, and I didn't even eat before meeting the Djigui [MMS] team, as soon as I started taking Djigui [MMS] my health returned, I have eaten a lot more and I gained weight.

—Woman in one of the MMS intervention groups

Many husbands participating in the group discussions expressed no concerns about their wives' taking MMS and were aware of its benefits in terms of ‘strengthening their blood’ to combat anaemia.

The difference [between IFA and MMS] is that Djigui [MMS] contains iron, vitamins, and micronutrients necessary for pregnant women. Djigui [MMS] strengthens health at the same time helps pregnant women in need of nutrition because it contains many foods [nutrients] that a woman needs.

—Influential family member

### 3.3 Perceived benefits of MMS versus IFA

Women in the IFA intervention group expressed appreciation for the benefits of the IFA supplements; however, women who had taken IFA in a previous pregnancy, then MMS in the most recent pregnancy, reported less nausea and vomiting, and greater appetite and weight gain for MMS compared to IFA. They also reported that IFA had a worse smell compared to MMS. Below are comments made by women.

Iron strengthens the blood, at the beginning of my pregnancy I was dizzy, as soon as I started taking the iron, I was relieved.

—Woman in in the IFA intervention group

For me, Djigui [MMS] is better than iron‐folic acid, because with the latter you have no appetite, while with Djigui [MMS] you have much more appetite.

—Woman in one of the MMS intervention groups

The smell of IFA is disturbing when I am taking it, and it makes me vomit, while Djigui [MMS] has no smell, and it is easy to take without problem.

—Woman in one of the MMS intervention groups

In addition, a few women claimed benefits from taking IFA and MMS that are not supported by scientific evidence and are beyond what was in the counselling guidelines. These claims included reduced or better‐regulated blood pressure and shorter labour. One woman asked if MMS might also function as a contraceptive. It was not clear if these extended benefits originated in their own experiences with the supplements, from discussions with other women, or if the midwives had communicated this information during counselling. Midwives were trained to give accurate information based on available scientific evidence and to address misperceptions when necessary.

### 3.4 Perceived barriers to taking supplements

The most significant concern raised about MMS was its effect on their baby's birthweight. Women questioned if, due to the many nutrients contained in the MMS, they would give birth to larger babies. Despite these concerns, many women took and adhered to MMS and reported a positive experience. For example, one woman stated the following.

In fact, I did not have any problem for this pregnancy, I noticed that I ate a lot during this pregnancy, and at birth the weight of the child was 3600 g, while for the previous pregnancy the weight of the child was at 2400 g.

—Woman in one of the MMS intervention groups

The concerns of women related to the negative side effects of taking MMS were echoed by midwives. One midwife stated the following.

For some women, they ask if Djigui [MMS] does not make them vomit too because it contains iron folic acid. For others they are afraid of having a big child because it has been said that the Djigui [MMS] protects the child against low birthweight.

—Midwife

The word ‘vitamin’ heightened the concern about larger babies, as vitamins promote fetal growth. To assuage this concern, project staff instructed midwives to use the word ‘Djigui’ or the Bambara term *fari.sinsilan* meaning ‘which strengthens the body’. However, the post‐intervention qualitative data collection showed that the concerns persisted and that even midwives continued to say ‘vitamin’.

### 3.5 Other perceived barriers to taking supplements

A couple of women in the IFA intervention group stated that they had not taken the supplements because it made them feel unwell or nauseous or the IFA had an unpleasant smell. Some women in the MMS intervention group who had already started IFA reported a resolution of nausea once they switched to MMS.

Because at the beginning of the pregnancy, it was the iron that I took, before meeting the Djigui [MMS] team. It was the iron that made me nauseous, I often hid this iron from my husband, telling him that I took it. But with Djigui [MMS] everything was fine.

—Woman in one of the MMS intervention groups

The standard of care in Mali is for IFA to be prescribed to pregnant women, who then must purchase it at a cost. In the context of this study, both MMS and IFA were provided free of cost. While that addressed a critical barrier to taking the supplements, for some, it also aroused suspicion. For example, women described the following.

The difference is that, for this pregnancy, I did not pay for medicine, and they gave it to me for free, [which I appreciated] especially with this poverty that is pervasive everywhere.

—Woman in one of the MMS intervention groups

In reality, I was given all the information about [Djigui], and also told me that it is a help for pregnant women, as I am a pharmacist, at first my husband was afraid because of the medicine being given free of charge…

—Woman in one of the intervention groups

Another barrier was that some women recounted difficulty in opening the bottles of MMS, which led a few women to skip taking MMS on some days. On the other hand, there was concern that using force to open the bottle could damage the seal around the top, in turn affecting the freshness of the MMS.

There was confusion about whether Djigui [MMS] was a nutrient, drug or medication. Despite midwives being encouraged to utilize Djigui [MMS] or *fari.sinsilan* instead of the vitamin when referring to MMS (as described above), it is clear from transcripts that pregnant women, their influential family members and ANC providers commonly referred to MMS as ‘fura’, a Bambara term meaning ‘a leaf’ that originally referred to herbal medicines, but now indicates both herbal and modern medicines or any sort of pill. The fact that Djigui [MMS] was presented in formal packaging rather than counted out and distributed loosely may have contributed to the misperception that Djigui [MMS] is a drug. Many family members stated that they believed Djigui [MMS] to be a drug and some family members sought to deter women from taking Djigui [MMS] out of concern for the harm that might result from taking drugs during pregnancy. A few husbands asserted that they did not want ‘Americanness’ to be forced on their wives, by them taking too many medications during their pregnancies. After seeking information from midwives in the health centres, husbands and other influential family members came to understand the purpose of the MMS and their concerns were assuaged.

We were informed by one [of] the midwives of the study that the husband of a woman had refused that his wife take Djigui [MMS]. The reasons given by this husband is that he is not familiar with Djigui [MMS], it is a drug from the Americans, and we already have the iron‐folate that his wife took in previous pregnancies, and therefore he does not want it.

—Midwife

### 3.6 Cues to action for taking supplements

Remembering to take MMS or IFA was commonly cited as a barrier to adherence. Many women confessed to being distracted throughout the day due to the many tasks they needed to accomplish. Spouses often reminded women to take the supplements and reassured them that problems encountered at the beginning would eventually be resolved. For example, women stated the following.

Djigui [MMS] pushed me to eat, and strengthened my health, which encouraged me a lot to take it easily.

—Woman in one of the MMS intervention groups

It was my husband who reminded me of taking the drug Djigui [MMS].

—Woman in one of the MMS intervention groups

In addition, pharmacy managers reported that they frequently gave women options on the best times of day to take MMS and IFA. In the two MMS intervention groups, midwives also handed out a calendar/tracking chart for women to note down each day that they took MMS. The calendar/tracking chart helped promote adherence by holding the women accountable for taking the supplements while allowing them autonomy over when to take them. While most women reported using the calendar/tracking sheet, some women expressed confusion on how to fill it out and others reported that it was a hardship for them to purchase a ballpoint pen, preferring a chart that would allow for capturing consumption by making holes rather than pen marks.

## 4 DISCUSSION

Perspectives of women, their husbands, midwives and pharmacy managers reveal that there is a belief that there are broad‐reaching benefits of MMS over IFA. However, misperceptions around MMS persist and continued efforts are needed to ensure they are addressed. Efforts to scale up MMS in Mali need to include messages that emphasize and promote the evidence‐based benefits of MMS, address barriers and concerns of pregnant women and influential family members and avoid endorsing false beliefs.

Our findings support those found by Aguayo et al. (2005) regarding women's ability to adhere to MMS when provided with consistent and easily understandable information. Women and providers concur that the logo, name, packaging, counselling, counselling aids and calendar/tracking sheet were instrumental in influencing the perceived benefit, uptake and adherence to MMS. There are, however, continued opportunities for improving counselling and counselling aids, and the training associated with their use.

While a variety of barriers exist, many appear to be minimal and can be mitigated through midwives' counselling and care. Therefore, focusing on ANC system strengthening in general is needed to ensure that women can get the best care depending on their pregnancy experience. Ensuring midwives have training and supportive supervision is critical to optimizing ANC delivery and MMS introduction.

As part of ANC strengthening, special consideration should be given to how to address midwives' limited time to provide comprehensive counselling around MMS during individual ANC encounters. Strategies to address this include developing mechanisms for further involving pharmacy managers in reinforcing messages, producing brief videos on specific topics (e.g. how to open and close the bottle or complete the calendar/tracking sheet) or adapting MMS counselling and associated materials for use in a group ANC setting where women share experiences and learn with their peers (Fuentes‐Rivera et al., 2020; Gaur et al., 2021; Grenier et al., 2022). Other community‐based avenues for building basic MMS awareness might include engaging community health workers—Community Health Agents (ASC) and Relais Communitaires (Relais)—to support knowledge diffusion and encourage adherence through proactive home visits and increased ANC contacts (Frontline Health Project, 2020; Kayentao et al., 2023).

Finally, family members, especially husbands, play a critical role in Mali and could be leveraged to increase MMS uptake and adherence. Development of feasible community‐based campaigns or incorporation of ANC and MMS messaging into existing social marketing and communication programmes such as health radio shows or through community outreach events could be combined with materials developed with and for husbands or other influential family members.

### 4.1 Study limitations

The qualitative evaluation was limited to data collection taking place after the full evaluation study was completed, and in‐depth interviews with women were only conducted post‐pregnancy. Collecting data at different or multiple time points during pregnancy or throughout study implementation would have allowed for a more robust understanding of how experiences, perceptions and opinions evolved over time for pregnant women and midwives. Study participants typically spoke either Bambara with many French words or French with many Bambara words, switching back and forth mid‐sentence. Many common terms in Bambara are highly nonspecific such as ‘fura’ which can mean (1) leaf of a tree or plant, (2) a traditional herbal remedy, (3) a modern medicine or (4) the micronutrient supplements provided in this study. Therefore, when women and family members were discussing their reactions to the supplements, at times it could be unclear what they were referring to. Additionally, while we intended to collect data from all midwives and pharmacists who had participated in the delivery of the intervention, we were unable to interview those who were not available during the scheduled FGD time.

This study did not look at how the supply chain and cost of supplements impact coverage and adherence to MMS. Although IFA is provided to pregnant women as part of a national policy, it is provided at a cost as part of a cost‐recovery plan. Both IFA and MMS were provided free of charge in this study. Efforts to introduce and scale up MMS require the government and other key stakeholders to ensure a transition plan that accounts for the MMS cost, acceptability and accessibility to all socioeconomic status strata.

While conducted in the context of the existing health system, programme staff were able to closely monitor implementation through direct observation of a select number of ANC visits and informal monitoring visits with ANC providers and study staff. As a result, many of the perceived barriers were identified and addressed during implementation and appear not to have affected high rates of adherence to supplement use among study participants. However, if MMS is scaled with less training or support, these same barriers are poised to impede the uptake and adherence of this critical intervention.

## 5 CONCLUSIONS

The findings from the qualitative evaluation indicate that MMS is highly acceptable within this community in Mali. Any transition from a national policy for IFA to MMS will need to include planning and budgeting for rolling out training on MMS counselling and associated resource materials and ongoing supportive supervision to ANC providers. The materials tested in this study may serve as an important starting point and provide a basis for the development of additional community‐based behaviour change strategies that may support MMS adherence for pregnant women. It is common across diverse contexts for women and their families to have concerns about taking supplements or medications during pregnancy and for misinformation about them to circulate. The codesign process described represents a short and feasible process to adapt packaging, supplements and messages to specific contexts. Other countries exploring the introduction of MMS may adopt a similar codesign process to develop a set of implementation strategies that strengthen the existing health system and enhance the beneficiary experience to optimize MMS uptake and adherence in their specific context.

## AUTHOR CONTRIBUTIONS

Aissata Ba, Adama Mamby Keita, Kounandji Diarra, Mahamane Djiteye, Baba Seydou Kanté, Moussa Coulibaly and Ousmane Dembele performed the research. Aissata Ba, Monica J. Fox, Adama Mamby Keita, Samba Sow, Shannon E. King, Lisa M. Noguchi, Kristen M. Hurley and Peter J. Winch designed the research study. Aissata Ba and Peter J. Winch analysed the data. Aissata Ba, Monica J. Fox and Peter J. Winch authored the paper. Lisa M. Noguchi, Adama Mamby Keita, Kristen M. Hurley and Pooja Sripad reviewed and provided important inputs into the final manuscript.

## CONFLICT OF INTEREST STATEMENT

K. M. H. and M. J. F., in addition to their affiliation at Johns Hopkins, are seconded to Vitamin Angel Alliance to provide technical support. Vitamin Angel Alliance provided the procurement and delivery of the product for the purpose of this study. The remaining authors declare no conflict of interest.

## Data Availability

Data that support the findings of this study are available from the corresponding author upon reasonable request.
